# Our Journal

**Published:** 1859-09

**Authors:** 


### EDITORIAL AND MISCELLANEOUS.

### OUR JOURNAL.

With this number commences a new volume, and we hope a recollection upon the part of subscribers of the unrequited labors of the Editor and the Publisher. We are willing to work, whether we get compensation or not, but not so is the Publisher, and we would again remind our friends that there is a large amount due upon the books, which we hope they will promptly remit.

The Editor, upon whom usually devolves the labor connected with the *Journal*, will be absent until after the issue of the next (October) No., but will leave it in competent hands. If, however, any want of attention should be manifest, he is confident that it will be overlooked and excused by those for whom he has labored, almost continuously, for the last four years.

We go away for *rest* of mind and body, without which we are clearly admonished that the last link of endurance will be sundered. We wish, however, to remark, in conclusion, that our work, to a great degree unrequited though it be, has been a "labor of love," and we wish to assure our friends that we feel the deepest interest in the future prosperity of the *Journal*, and if spared to return to our labors, expect to bring greater energy to its advancement.

